# Dr. Waterman on Spectroscopic Analysis

**Published:** 1878-03

**Authors:** 


### EDITORIAL, ETC.

---

*Dr. Waterman on Spectroscopic Analysis.*—We print elsewhere an exceedingly interesting and instructive article from the pen of Dr. Sigismund Waterman, of New York City, on *Spectroscopic Analysis*, and especially the analysis of blood under the action of anæsthetics. The subject is a new one, comparatively, and although but little studied in this country, is much thought of among scientific men in Europe, and it will doubtless lead to important results. The uses of the spectroscope in diagnosis of ovarian and other tumors is already recognized and utilized by Marion Sims and others, and it will no doubt come to be a common method of diagnosis in many cases not only of this sort but of blood poisoning and determination of now obscure points in medical jurisprudence. The action of nitrous oxide on the blood, if correctly stated by Dr. Waterman, is a subject of grave importance, and if true should be known not only by the dental and medical professions, but by the public generally.

Dr. Waterman is inviting correspondence with dentists and medical men on the subject of the " after effects of anæsthetics," and as observed by them, and hopes to obtain some valuable statistics. Without consulting him, we take the liberty of giving his address, and solicit for him correspondence on the above subject. Address Dr. S. Waterman, 103 West 49th Street, New York City.

Dr. Waterman has been for the past few days in Baltimore, and delivered several lectures before the Medical and Dental Schools here; the substance of those addresses are embraced in the paper we print. They were highly appreciated by those who heard them.

In this connection the paper we publish by Dr. J. J. Chisholm, Professor of Eye and Ear Diseases, in the Maryland University School of Medicine, will be read with great interest. Dr. Chisholm, it will be seen takes extreme ground in favor of chloroform, and considers that it lessens rather than increases the dangers attendant upon operations on a class of patients whose organic diseases are usually considered as contra-indicating its use. The paper is a bold one, and although not prepared to dispute its positions, we cannot but feel it our duty to caution our brethren of the dental profession against the free use of an agent, which, whatever may be its harmlessness and efficiency in the hands of medical men, has certainly a black record in the archives of dentistry. H.

